# Placental circadian lincRNAs and spontaneous preterm birth

**DOI:** 10.3389/fgene.2022.1051396

**Published:** 2023-01-11

**Authors:** Guoli Zhou, Raina N. Fichorova, Claudia Holzman, Bin Chen, Chi Chang, Eric P. Kasten, Hanne M. Hoffmann

**Affiliations:** ^1^ Clinical and Translational Sciences Institute, Michigan State University, East Lansing, MI, United States; ^2^ Department of Obstetrics, Gynecology and Reproductive Biology, Brigham and Women’s Hospital, Harvard Medical School, Boston, MA, United States; ^3^ Department of Epidemiology and Biostatistics, Michigan State University, East Lansing, MI, United States; ^4^ Department of Pediatrics and Human Development, Michigan State University, East Lansing, MI, United States; ^5^ Department of Radiology, Michigan State University, East Lansing, MI, United States; ^6^ Department of Animal Science, College of Agriculture and Natural Resources, Michigan State University, East Lansing, MI, United States

**Keywords:** preterm (birth), molecular clock, lincRNA, transcriptional co-alteration, GSVA analysis

## Abstract

Long non-coding RNAs (lncRNAs) have a much higher cell- and/or tissue-specificity compared to mRNAs in most cases, making them excellent candidates for therapeutic applications to reduce off-target effects. Placental long non-coding RNAs have been investigated in the pathogenesis of preeclampsia (often causing preterm birth (PTB)), but less is known about their role in preterm birth. Preterm birth occurs in 11% of pregnancies and is the most common cause of death among infants in the world. We recently identified that genes that drive circadian rhythms in cells, termed molecular clock genes, are deregulated in maternal blood of women with spontaneous PTB (sPTB) and in the placenta of women with preeclampsia. Next, we focused on circadian genes-correlated long intergenic non-coding RNAs (lincRNAs, making up most of the long non-coding RNAs), designated as circadian lincRNAs, associated with sPTB. We compared the co-altered circadian transcripts-correlated lincRNAs expressed in placentas of sPTB and term births using two published independent RNAseq datasets (GSE73712 and GSE174415). Nine core clock genes were up- or downregulated in sPTB *versus* term birth, where the *RORA* transcript was the only gene downregulated in sPTB across both independent datasets. We found that five circadian lincRNAs (*LINC00893*, *LINC00265*, *LINC01089*, *LINC00482*, and *LINC00649*) were decreased in sPTB vs term births across both datasets (*p* ≤ .0222, FDR≤.1973) and were negatively correlated with the dataset-specific clock genes-based risk scores (correlation coefficient r = −.65 ∼ -.43, *p* ≤ .0365, FDR≤.0601). Gene set variation analysis revealed that 65 pathways were significantly enriched by these same five differentially expressed lincRNAs, of which over 85% of the pathways could be linked to immune/inflammation/oxidative stress and cell cycle/apoptosis/autophagy/cellular senescence. These findings may improve our understanding of the pathogenesis of spontaneous preterm birth and provide novel insights into the development of potentially more effective and specific therapeutic targets against sPTB.

## 1 Introduction

Preterm birth (PTB) is a major contributor to infant mortality, morbidity, and later-life handicapping conditions (Chang et al., 2013). The global PTB rate is about 11% (Chang et al., 2013; Middleton et al., 2018). Approximately 40%–45% of PTBs are spontaneous preterm labor, 25%–30% are related to preterm premature rupture of membranes (PPROM), and 30%–35% are attributed to medically indicated preterm deliveries (Goldenberg et al., 2008). Both spontaneous preterm labor and PPROM are often combined and designated as spontaneous preterm birth (sPTB) (Goldenberg et al., 2008). Many factors have been associated with an increased risk of sPTB such as impaired placenta function, maternal age and race, previous PTB history, vaginal bleeding during pregnancy, shortened cervix, smoking, and maternal chronic conditions (e.g., high blood pressure, diabetes, autoimmune disease, and depression) (Goldenberg et al., 2008). Despite intensive research, the pathophysiology and the relevant molecular mechanisms of sPTB remain unclear, which might explain why we lack the effective means of sPTB prediction/prevention.

The placenta is the anatomic interface between mother and fetus and plays a critical role in regulating nutrient supply to the fetus and producing hormones that control both fetal and maternal physiology during pregnancy (Illsley, 2011; McNamara and Kay, 2011). Therefore, the placenta has become a promising target for investigating the etiology and/or pathophysiology of sPTB and subsequently for developing precision diagnosis and therapeutics of sPTB. Studies on global placental gene expression profiling linked to sPTB are emerging and have provided promising novel insights into the etiology and pathogenesis of sPTB (Ackerman et al., 2016; Huusko et al., 2021; Lien et al., 2021). One possible mechanism is that the placental “molecular clock”, a cell endogenous time-keeping mechanism, might be required for female reproductive success. Studies in mice have shown that loss of molecular clock function reduces reproductive success (Miller et al., 2004; Kennaway et al., 2012; Sen and Hoffmann, 2020; Yaw et al., 2020; Hoffmann et al., 2021), and is associated with increased risk of mis-timed birth and dystocia (Ratajczak et al., 2012; Miller and Takahashi, 2014). Our recent studies in human placenta indicate that co-alterations of the molecular clock mRNAs *NR1D2*, *CLOCK* and *PER3* are significantly associated with term preeclampsia (Zhou et al., 2022), whereas co-alteration of *CLOCK* and *CRY2* at the mRNA levels in second trimester maternal blood are significantly associated with an increased risk of sPTB (Zhou et al., 2021a). However, the expression pattern of core molecular clock genes in placentas of sPTB vs term births, as well as the underlying mechanism(s) are still unknown.

LncRNAs are a class of endogenous, non-protein coding RNAs longer than 200 nucleotides. Long intergenic non-coding RNAs (lincRNAs) represent the major type of lncRNAs. The function of lncRNAs include chromatin modification, transcriptional regulation, and RNA processing (Fernandes et al., 2019; Statello et al., 2021). Expression of many lncRNAs (including lincRNAs) have much higher cell- and/or tissue-specificity compared to mRNAs, making them excellent candidates for therapeutic applications to reduce off-target effects (Arun et al., 2018). Growing evidence indicates that lncRNAs play an important role in the pathogenesis of various chronic diseases (Yarani et al., 2018; Das et al., 2020; Zhou et al., 2021b). Placental lncRNAs specifically have been implicated in the pathogenesis of preeclampsia (a disease often leading to PTB), e.g., regulating the proliferation, invasion, and migration of placental trophoblast cells (Chen et al., 2020; Monteiro et al., 2021). However, to date, we are aware of only one published, small (10 PPROM cases) clinical study investigating the relationship between placental lncRNAs and PTB (Luo et al., 2013). Thus, more rigorous studies are needed to further clarify the potential role of placental lncRNAs in relation to PTB. On the other hand, studies with mouse models have revealed that a large number of lncRNAs are regulated by the molecular clock (Fan et al., 2017; Qi et al., 2020; Wang et al., 2022). However, to date, no reports on placental circadian genes-related lincRNAs associated with PTB including sPTB have been found.

In this study, we compared the co-altered circadian transcripts-correlated lincRNAs, designated as circadian lincRNAs, in placentas of sPTB *versus* term birth groups using two independent RNAseq datasets (GSE73712 and GSE174415) from the National Center for Biotechnology Information (NCBI) Gene Expression Omnibus (GEO) database. We selected 14 core circadian transcripts (*ARNTL*, *ARNTL2*, *CLOCK*, *CRY1*, *CRY2*, *NPAS2*, *NPAS3*, *NR1D1*, *NR1D2*, *PER1*, *PER2*, *PER3*, *RORA*, and *TIMELESS*) based on our previous study (Zhou et al., 2022) and the detectability of their mRNAs in both datasets. We also analyzed the circadian lincRNAs-correlated and sPTB-associated biological pathways using the Gene Set Variation Analysis (GSVA) (Zhou et al., 2022).

## 2 Materials and methods

### 2.1 Study design, selection of patients, and demographics

The GSE73712 was a set of RNAseq data of human placentas from 10 term births and 20 sPTBs, among which half of both sPTB and term births were sampled from the villous trophoblast region and another half from the decidual basalis region of placentas (Ackerman et al., 2016). The samples in this data set excluded those with chromosomal aneuploidy, fetal structural abnormalities, multi-fetal gestations, uterine contractions at term, known maternal medical conditions (e.g., chronic hypertension, preeclampsia, diabetes, thrombophilias, thyroid disease), viral infection (e.g., human immunodeficiency virus, hepatitis B virus), and fetal heart rate abnormalities (Ackerman et al., 2016). The sPTB group was a mixture of preterm labor (defined as the presence of regular uterine contractions and documented cervical effacement and/or dilatation in patients <37 weeks of gestational age) and PPROM (confirmed by vaginal “pooling”, and positive “nitrazine” or “ferning” tests) (Ackerman et al., 2016). The race/ethnicity of the study population is unclear in the original research (Ackerman et al., 2016).

The GSE174415 dataset was derived from 32 African American women’s placental tissues (16 sPTB and 16 term births) (Lien et al., 2021). Placenta samples were collected from mid-placenta near the cord insertion on the fetal side (Lien et al., 2021). sPTB was defined as preterm labor (defined as regular contractions and cervical dilation) or PPROM occurring between >20 and <37 weeks of gestational age (Lien et al., 2021). Women with multiple gestations, fetal chromosomal abnormalities, major fetal anomalies, intrauterine fetal demise, intrauterine growth restriction, clinical chorioamnionitis, induction of labor, elective cesarean delivery, gestational diabetes, and gestational hypertension or preeclampsia were excluded (Lien et al., 2021).

Individual-level information about demographic and clinical characteristics associated with placental specimens were not available in both datasets. All sequenced reads in. fastq files were generated by using the Illumina HiSeq system, aligned to the reference human genome (hg38), and curated as gene-level raw (i.e., non-normalized) read counts in both studies (Ackerman et al., 2016; Lien et al., 2021). We re-analyzed the gene-level raw read counts in both datasets (see below for details).

### 2.2 Bioinformatics and statistical analyses

**FIGURE 1:**
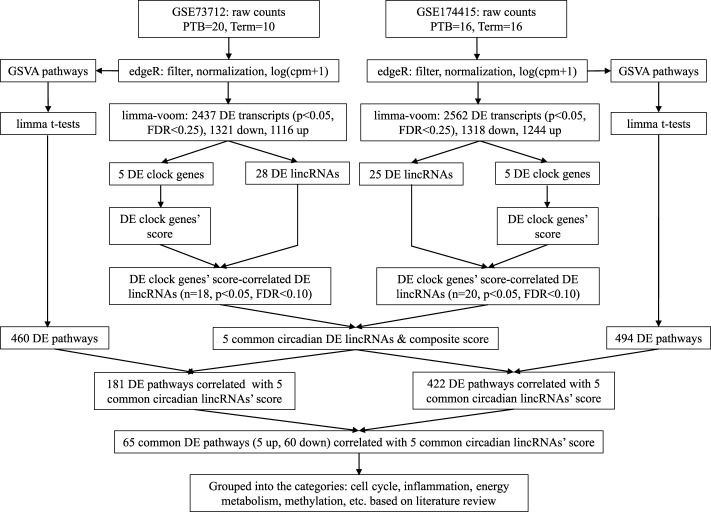
Analytic pipeline to identify placental circadian lincRNAs and pathways in sPTB vs term birth.

Figure 1 is a bioinformatics pipeline that we developed to identify sPTB-associated circadian lincRNAs and their correlated pathways with two independent datasets. Before starting with this pipeline, the duplicates of the transcript ID or name were removed by keeping the highest value. Then, the two datasets were filtered by the setting of the raw counts>10 (i.e., counts-per-million (cpm) > .5) in at least 70% samples followed by log (cpm+1)-transformation and trimmed mean of the M-values (TMM) normalization using the *edgeR* package (Robinson et al., 2010). The normalized data were further *limma-voom*-transformed (Law et al., 2014) and then used to compute moderated t-statistics with eBayes () function in *limma* R package for detecting differentially expressed (DE) transcripts (Ritchie et al., 2015). Next, we retrieved 14 core circadian transcripts (*ARNTL*, *ARNTL2*, *CLOCK*, *CRY1*, *CRY2*, *NPAS2*, *NPAS3*, *NR1D1*, *NR1D2*, *PER1*, *PER2*, *PER3*, *RORA*, and *TIMELESS*) based on our previous study (Zhou et al., 2022) and the detectability of their mRNAs in both datasets. The mRNA levels (log (cpm+1)) of all these circadian genes in placentas were summarized as means with standard deviations (SDs) together with the moderated t statistics, *p*-values, and false discovery rates (FDRs) that were extracted from the output of the *limma-voom* DE transcripts’ analysis. The cut-off values for statistical significance were set as *p* < .05 and FDR q < .25 (Drizik et al., 2020). In order to obtain the DE lincRNAs that are correlated with the identified DE clock genes as a whole, we used an unweighted linear combination of the DE clock transcripts to generate a composite score (Zhou et al., 2022), designated as “DE clock genes-based risk score” (Figure 1). The levels of DE lincRNAs were extracted from the output of the *limma-voom* analysis of the same filtered and normalized RNAseq datasets and then the correlations of the DE clock genes-based risk score with the DE lincRNAs were examined using the Pearson correlation method (Figure 1). The significant DE lincRNAs correlated with the DE clock genes-based risk score were determined with the cut-off significance values of *p* < .05 and FDR<.10 (Zhou et al., 2020), which were designated as sPTB-associated circadian lincRNAs. Finally, we overlapped the two pools of significant sPTB-associated circadian lincRNAs identified from the two independent datasets to generate common significant sPTB-associated circadian lincRNAs across the two datasets, designated as circadian lincRNAs (Figure 1). In order to explore whether these five circadian lincRNAs have functional similarities, we retrieved their genomic locations and transcript sequences from the GeneCards Suite databases (Stelzer et al., 2016) and NCBI Reference Sequence (RefSeq) database (NCBI Entrez Gene) (O’Leary et al., 2016) followed by a multiple sequence alignment to find the transcript sequence identity among these five lincRNAs *via* a web program - Clustal Omega (https://www.ebi.ac.uk/Tools/msa/clustalo/) (Madeira et al., 2022). In order to capture the variation in these five circadian lincRNAs between sPTB and term births, we performed a principal component analysis (PCA) of all samples using R prcomp () and autoplot () functions.

Next, the Gene Set Variation Analysis (GSVA) analysis was conducted with two datasets (log (cpm+1)-transformed) to explore the biological pathways that are related to the identified circadian lincRNAs, respectively, by referring to our previous method (Zhou et al., 2022) (Figure 1). Compared to the classical gene set enrichment analysis, the GSVA method increases the statistical power to detect subtle changes of pathway activity over a sample population (Hänzelmann et al., 2013). We used the pathway sub-collection - c2. cp.v7.5.1. symbols.gmt in the Molecular Signatures Database (MSigDB). The differential pathways between sPTB and term births were examined using *limma* R package (Ritchie et al., 2015) with the cut-offs of *p* < .05 and FDR<.25 (Figure 1). Similarly, in order to obtain the DE pathways that are correlated with five common circadian DE lincRNAs as a whole, we used an unweighted linear combination of the five common circadian DE lincRNAs to generate a composite score (Zhou et al., 2022), designated as “common circadian DE lincRNAs-based risk score” (Figure 1). Then, the correlations between the circadian lincRNAs-based risk score and the sPTB-associated pathways were analyzed using the Pearson correlation method (*p* < .05 and FDR<.10) (Figure 1). Finally, two pools of sPTB-associated pathways correlated with common circadian lincRNAs-based risk score were overlapped to generate common pathways (Figure 1).

All data management and statistical analyses described above were conducted with R (R Development Core Team) and SAS v9.4 (SAS Institute, Cary, North Carolina).

## 3 Results

### 3.1 Differentially expressed (DE) circadian genes in placental tissue between sPTB and term groups

After removing the transcripts with very low counts across all the libraries, we had a total of 15,929 and 14,195 transcripts remaining in the GSE73712 and GSE174415 datasets, respectively, which included 14 core molecular clock genes as well as 175 and 106 lincRNAs, respectively (data not shown). *Limma-voom* moderated t-tests revealed that 2,347 and 2562 DE transcripts were detected from the GSE73712 (1,321 down- and 1,116 upregulated) and GSE174415 (1,318 down- and 1,244 upregulated), respectively (Figure 1). Among these DE transcripts, nine core molecular clock genes were differentially expressed between the two groups (*NPAS2*, *NR1D1*, *NR1D2*, *PER3*, and *RORA* in GSE73712; *ARNTL*, *CRY1*, *NPAS3*, *PER2*, and *RORA* in GSE174415) (Figure 1; Table 1).

**TABLE 1:** Differential expressions of detectable circadian gene transcripts in placenta between sPTB and term births.

Gene	Term	sPTB	Moderated t	*p*	FDR
	Mean (SD)	Mean (SD)			
GSE73712 dataset (*n* = 10 for Term, *n* = 20 for sPTB)					
*ARNTL*	3.45 (.32)	3.62 (.33)	1.25	.2203	.5278
*ARNTL2*	5.49 (.25)	5.42 (.27)	−1.03	.3109	.6158
*CLOCK*	6.59 (.17)	6.60 (.14)	−.66	.5116	.7658
*CRY1*	5.01 (.17)	4.90 (.15)	−2.01	.0533	.2868
*CRY2*	4.92 (.20)	5.04 (.23)	.92	.3656	.6642
*NPAS2*	4.97 (.36)	4.65 (.33)	−3.14	.0037	.0841
*NPAS3*	1.64 (.85)	1.19 (.65)	−1.59	.1231	.4032
*NR1D1*	1.93 (.51)	1.22 (.93)	−2.53	.0169	.1750
*NR1D2*	6.64 (.40)	6.12 (.57)	−2.98	.0056	.1003
*PER1*	4.71 (.37)	5.22 (.67)	2.06	.0484	.2759
*PER2*	5.13 (.53)	5.00 (.31)	−1.02	.3145	.6193
*PER3*	3.48 (.43)	3.00 (.43)	−3.37	.0020	.0615
*RORA*	6.50 (.41)	6.04 (.34)	−3.51	.0014	.0535
*TIMELESS*	4.35 (.18)	4.51 (.43)	.83	.4148	.7028
*GSE174415 dataset (n = 16 for Term, n = 16 for sPTB)*					
*ARNTL*	3.04 (.43)	3.35 (.38)	2.65	.0120	.1475
*ARNTL2*	5.67 (.21)	5.69 (.29)	.17	.8630	.9393
*CLOCK*	6.17 (.25)	6.21 (.22)	.44	.6639	.8366
*CRY1*	5.19 (.17)	4.91 (.23)	−3.05	.0043	.0975
*CRY2*	4.35 (.30)	4.27 (.17)	−.98	.3319	.6087
*NPAS2*	4.52 (.50)	4.33 (.40)	−1.51	.1408	.4114
*NPAS3*	.71 (.57)	.28 (.56)	−2.19	.0357	.2257
*NR1D1*	.27 (.76)	.12 (1.04)	−.54	.5932	.7987
*NR1D2*	6.09 (.46)	6.08 (.33)	−.12	.9075	.9600
*PER1*	3.54 (.62)	3.41 (.44)	−.75	.4606	.7102
*PER2*	4.99 (.30)	4.55 (.39)	−3.65	.0008	.0498
*PER3*	2.17 (.62)	2.30 (.50)	.69	.4950	.7333
*RORA*	6.33 (.34)	6.04 (.42)	−2.28	.0292	.2075
*TIMELESS*	3.44 (.46)	3.68 (.33)	1.89	.0665	.2954

*Cut-off values for statistical significance were set as *p* < .05, FDR <.25.

**FIGURE 2:**
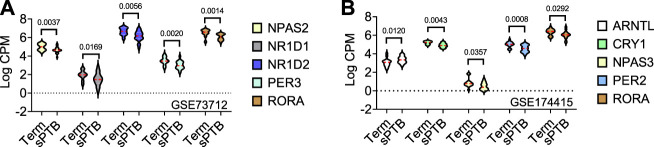
Violin plots of five differentially expressed core clock genes in sPTB vs Term in two datasets. Violin plots for **(A)** GSE73712, and **(B)** GSE174415. The red loosely dashed line in the center of each violin box represents the mean and the white dotted lines in the center represent the confidence limits for the population mean. All *p*-values on the top of the bars were generated by the moderated *t*-test in limma R package.

In the GSE73712 the average expressions of DE core molecular clock gene transcripts (*NPAS2*, *NR1D1*, *NR1D2*, *PER3*, and *RORA*) were lower in sPTB than in term birth (Table 1; Figure 2). In contrast, in the GSE174415 dataset, the mean of *ARNTL* gene transcript was significantly higher in sPTB than in term, while four other molecular clock genes (*CRY1*, *NPAS3*, *PER2*, and *RORA*) were significantly decreased in sPTB vs term (Table 1; Figure 2). Among these 9 DE clock genes, only the *RORA* transcript was robustly downregulated in sPTB vs term across both independent datasets (Table 1). The remaining circadian gene transcripts were not significantly associated with sPTB in both datasets (Table 1; Figure 2).

### 3.2 Common circadian DE lincRNAs in placental tissue between sPTB and term groups across two datasets

As described above, after removing the transcripts with very low counts across all the libraries, there were 175 and 106 lincRNAs detectable in GSE73712 and GSE174415 datasets, respectively (data not shown). To adapt the data for downstream moderated *t*-test, the *Limma-voom* was applied. The results revealed that 28 and 25 DE lincRNA transcripts were detected from the GSE73712 and GSE174415, respectively (Figure 1).

To find the circadian DE lincRNAs, we first linearly combined the levels of the five molecular clock gene transcripts identified in each dataset, designated as a combined clock genes-based risk score (Figure 1). Specifically, for a “protective” clock gene (i.e., the expression of the gene is downregulated in sPTB), we used this “protective” gene’s maximum value among all samples minus this gene’s expression value for a given individual sample to represent the contribution of this ‘protective’ gene in the defined clock genes-based risk score (Zhou et al., 2022). Based on the identified DE clock genes in Table 1 and their corresponding maximum values, the clock genes-based risk score equaled [(6-*NPAS2*) + (3-*NR1D1*) + (8-*NR1D2*) + (5-*PER3*) + (7-*RORA*)] for GSE73712 and [*ARNTL* + (6-*CRY1*) + (2-*NPAS3*) + (6-*PER2*) + (7-*RORA*)] for GSE174415, respectively (Table 2, see footnote). Then, we applied the Pearson correlation method to analyze the correlations between the clock genes-based risk score and DE lincRNAs in the two datasets, separately (Figure 1). The results demonstrated there were 18 and 20 DE lincRNAs significantly correlated with the molecular clock genes-based risk score in GSE73712 and GSE174415 (*p* < .05 and FDR<.10), respectively (Figure 1). Finally, we obtained five common circadian DE lincRNAs (*LINC00893*, *LINC00265*, *LINC01089*, *LINC00482*, and *LINC00649*) across the two datasets by overlapping these two pools of the circadian DE lincRNAs (Figure 1; Table 2).

**TABLE 2:** Five common lincRNAs associated with both sPTB (vs Term) and co-alteration of clock gene transcripts in placenta across two independent datasets.

LincRNA	Term (n = 10)	sPTB (n = 20)	Moderated t for DE lincRNAs			Clock genes-based risk score^a^ correlation		
	Mean (SD)	Mean (SD)	t	p^b^	FDR^b^	r	p^c^	FDR^c^
GSE73712 (Term = 10, sPTB = 20)								
*LINC00893*	2.79 (.36)	2.35 (.52)	−3.12	.0039	.0855	−.64 (−.81, −.37)	<.0001	.0014
*LINC00265*	3.53 (.24)	2.98 (.33)	−5.30	<.0001	.0059	−.61 (−.80, −.32)	.0002	.0019
*LINC01089*	3.96 (.23)	3.79 (.22)	−2.41	.0222	.1973	−.50 (−.73, −.17)	.0040	.0144
*LINC00482*	4.82 (.51)	4.34 (.37)	−3.31	.0024	.0670	−.45 (−.70, −.10)	.0124	.0316
*LINC00649*	3.52 (.27)	3.11 (.43)	−3.33	.0023	.0656	−.38 (−.65, −.03)	.0365	.0601
GSE174415 (Term = 16, sPTB = 16)								
*LINC00893*	2.85 (.94)	1.86 (.93)	−3.36	.0019	.0694	−.50 (−.72, −.19)	.0030	.0075
*LINC00265*	3.07 (.59)	2.55 (.70)	−2.80	.0083	.1274	−.43 (−.67, −.09)	.0144	.0234
*LINC01089*	2.39 (.38)	1.80 (.44)	−4.19	.0002	.0279	−.65 (−.81, −.39)	<.0001	.0025
*LINC00482*	4.41 (.43)	3.86 (.49)	−3.11	.0037	.0914	−.51 (−.73, −.19)	.0027	.0075
*LINC00649*	3.54 (.41)	3.02 (.37)	−3.93	.0004	.0368	−.45 (−.69, −.12)	.0091	.0175

^a^ For GSE73712 dataset, the clock genes-based risk score = (6-*NPAS2*) + (3-*NR1D1*) + (8-*NR1D2*) + (5-*PER3*) + (7-*RORA*) based on the identified differentially expressed (DE) clock genes in Table 1 and their corresponding maximum values. For GSE174415 dataset, the clock genes-based risk score = *ARNTL* + (6-*CRY1*) + (2-*NPAS3*) + (6-*PER2*) + (7-*RORA*) based on the identified DE, clock genes in Table 1 and their corresponding maximum values.

^b^ Cut-off values for moderated t tests were set as *p* < .05 and FDR<.25.

^c^ Cut-off values for multiple Pearson correlations were set as *p* < .05 and FDR<.10.

**FIGURE 3:**
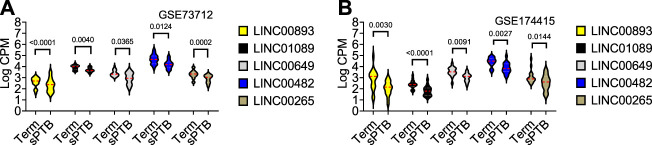
Violin plots of the expressions of five common circadian DE lincRNAs in sPTB vs Term in two datasets. Violin plots for **(A)** GSE73712, and **(B)** GSE174415. The red loosely dashed line in the center of each violin box represents the mean and the white dotted lines in the center represent the confidence limits for the population mean. All *p*-values on the top of the bars were generated by the moderated *t*-test in limma R package.

**FIGURE 4:**
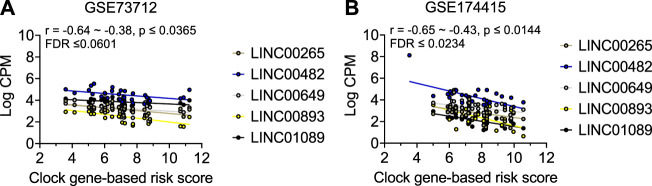
Visualization of the correlations between clock genes-based risk score and five common circadian DE lincRNAs in two datasets. Linear correlation between the clock genes-based risk score with each of the 5 DE lincRNA for **(A)** GSE73712 dataset, **(B)** GSE174415. The dots represent raw data points, and the lines represent linear regression lines for the relationships of clock genes-based risk score with each of five common circadian DE lincRNAs. The clock genes-based risk score = [(6-NPAS2) + (3-NR1D1) + (8-NR1D2) + (5-PER3) + (7-RORA)] for GSE73712 and [ARNTL + (6-CRY1) + (2-NPAS3) + (6-PER2) + (7-RORA)] for GSE174415, respectively.

As shown in Table 2 and Figure 3, the average expressions of all five circadian lincRNA transcripts (*LINC00893*, *LINC00265*, *LINC01089*, *LINC00482*, and *LINC00649*) in both datasets were significantly lower in sPTB than in term birth. According to Table 1, eight out of nine clock genes in at least one of two datasets were significantly decreased in sPTB vs term, which were accompanied by the significant decrease of all five circadian lincRNAs (Table 2). Using the dataset-specific clock genes-based risk score, we found that there were significantly negative correlations between this risk score and these individual circadian DE lincRNAs in both datasets (Figure 4). Further analysis with the multiple sequence alignment revealed that there are about 41.1%–53.1% sequence identity among these five circadian lincRNAs (Supplementary Table S1). PCA analysis of these five circadian lincRNAs in the two datasets reveals that there are two major clusters: one clusters between 0 and .3 or .2 on *x*-axis (PC1) and the other clusters between −.3 and 0 on *x*-axis (Supplementary Figure S1). The PCA plot also demonstrates that both groups have a substantial variability in the expression of five circadian lincRNAs across individual samples, i.e., the heterogeneity of these five circadian lincRNAs in the samples (Supplementary Figure S1).

### 3.3 Biological pathways predicted to be enriched by the five common circadian lincRNAs in sPTB vs term births across two datasets

In order to observe the global biological pathways that are associated with sPTB vs term births, we converted the log (cpm+1)-transformed transcript matrix into a GSVA pathway score matrix at the individual sample level using the GSVA method (Hänzelmann et al., 2013), followed by the analysis of the sPTB-enriched pathways using the classical *limma* R package (Law et al., 2014) for two datasets, separately (Figure 1). The results indicated that there were 460 and 494 pathways significantly associated with sPTB (data not shown) (Figure 1).

**FIGURE 5:**
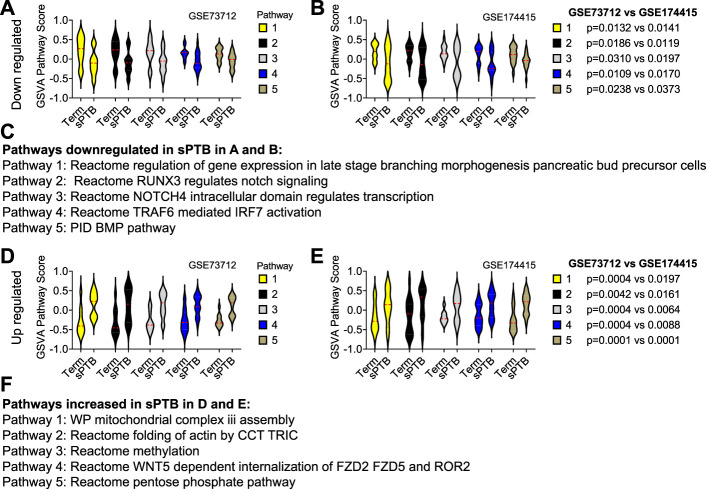
Violin plots of top 5 decreased or increased pathway scores in sPTB vs Term in two datasets. Violin plots of the top 5 decreased pathways for **(A)** GSE73712, and **(B)** GSE174415, and the top five increased pathways for **(D)** GSE73712, and **(E)** GSE174415. The names of the predicted **(C)** down and **(F)** upregulated pathways identified in **(A)**, **(B)** and **(D)**, **(E)**, respectively. The red loosely dashed line in the center of each violin box represents the mean and the white dotted lines in the center represent the confidence limits for the population mean. The pathway scores were generated by the GSVA R package. All *p*-values on the top of the bars were generated by the moderated *t*-test in limma R package (Tables S2 and S3).

**FIGURE 6:**
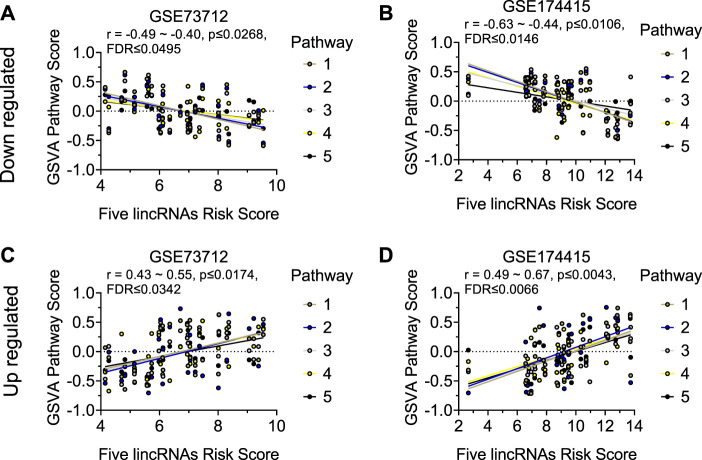
Visualization of the correlations between five circadian lincRNAs-based risk score and top 5 decreased or increased pathway scores in two datasets. Linear correlation between the five circadian lincRNAs-based risk score and each of top 5 decreased pathway scores for **(A)** GSE73712 dataset, **(B)** GSE174415. Linear correlation between the five circadian lincRNAs-based risk score and each of top five increased pathways for **(C)** GSE73712 dataset, **(D)** GSE174415. The dots represent raw data points, and the lines represent linear regression lines for the relationships of five lincRNAs-based risk score with each of top five pathways. The five lincRNAs-based risk score = [(5-LINC01089) + (6-LINC00482) + (4-LINC00893) + (5-LINC00649) + (4-LINC00265)] for GSE73712, and [(4-LINC01089) + (5-LINC00482) + (5-LINC00893) + (5-LINC00649) + (5-LINC00265)] for GSE174415, respectively. The pathway scores were generated by the GSVA R package. The names of the identified up and downregulated pathways are indicated in Figures 5C, F, respectively.

Similar to the clock genes-based risk score, we also linearly combined the levels of the five circadian lincRNA transcripts in the two datasets, designated as circadian lincRNAs-based risk score, which equaled [(5-*LINC01089*) + (6-*LINC00482*) + (4-*LINC00893*) + (5-*LINC00649*) + (4-*LINC00265*) for GSE73712, and (4-*LINC01089*) + (5-*LINC00482*) + (5-*LINC00893*) + (5-*LINC00649*) + (5-*LINC00265*) for GSE174415 (Table 2). Then, we applied the Pearson correlation method to analyze the correlations between the five circadian lincRNAs-based risk score and DE pathways in the two datasets, separately (Figure 1). The results revealed that there were 181 and 422 DE pathways significantly correlated with the five circadian lincRNAs-based risk score in GSE73712 and GSE174415 (*p* < .05 and FDR<.10), respectively (Figure 1). Finally, we overlapped the two pools of the pathways and obtained a total of 65 pathways (5 down- and 60 upregulated) that were enriched by the five circadian lincRNAs in sPTB across the two datasets (Supplementary Tables S2, S3). As shown in Figure 5, the identified top 5 decreased or increased pathways in sPTB in the GSE73712 dataset were also observed to be significantly decreased or increased in sPTB, respectively, in the GSE174415 dataset. Furthermore, these decreased or increased pathways were observed to be negatively or positively correlated with the five circadian lincRNAs-based risk score, respectively, in both datasets (Figure 6).

Based on a literature review and our previous studies (Zhou et al., 2022), we manually searched the published research papers (regardless of cell origin and mammalian species) to examine possible links between each of these 65 pathways and at least one of the physiological/pathophysiological processes including “cell cycle”, “apoptosis”, “autophagy”, “cellular senescence”, “immune”, “inflammation”, “oxidative stress”, “energy metabolism”, “protein synthesis”, and/or “methylation” in the PubMed database. The results suggested that out of the 65 pathways: about 31 can be linked to the cell cycle, apoptosis, autophagy, and cellular senescence; 34 relate to immune, inflammation, and oxidative stress; eight relate to energy metabolism and protein synthesis; and five are associated with methylation (Table 3). Some of these pathways can be linked simultaneously to multiple processes described above (Table 3).

**TABLE 3:** Common pathways that are enriched by both five circadian lincRNAs in placenta and sPTB (vs Term) across two independent datasets.

Five common circadian LincRNAs-Based risk Score^a^-Correlated pathway	GSE73712			GSE174415		
	r (95% CL)	p^b^	FDR^b^	r (95% CL)	P^b^	FDR^b^
Decreased pathways in sPTB						
^c^ ^,^ ^d^PID BMP Pathway	−.44 (−.69, −.09)	.015	.0304	−.44 (−.69, −.11)	.0106	.0146
^c^REACTOME NOTCH4 intracellular domain regulates transcription	−.40 (−.67, −.05)	.0268	.0495	−.49 (−.71, −.17)	.0042	.0064
^c^ ^,^ ^d^Reactome regulation of gene expression in late stage branching morphogenesis pancreatic bud precursor cells	−.49 (−.73, −.16)	.0049	.0122	−.61 (−.79, −.34)	.0001	.0003
^c^ ^,^ ^d^Reactome RUNX3 regulates notch signaling	−.46 (−.70, −.12)	.0102	.0221	−.62 (−.80, −.35)	<.0001	.0003
^c^Reactome TRAF6 mediated IRF7 activation	−.41 (−.67, −.06)	.0238	.0449	−.63 (−.80, −.36)	<.0001	.0003
Increased pathways in sPTB						
^c^BIOCARTA COMP pathway	.55 (.23, .76)	.0015	.0045	.49 (.17, .72)	.0040	.0062
^c^ ^,^ ^d^BIOCARTA EIF4 pathway	.49 (.15, .72)	.0057	.0134	.40 (.06, .66)	.0219	.0277
^d^BIOCARTA TFF pathway	.55 (.23, .76)	.0014	.0043	.35 (.00, .62)	.0496	.0581
^d^KEGG Alzheimers disease	.43 (.08, .68)	.0183	.0358	.72 (.49, .85)	<.0001	.0003
^c^KEGG beta alanine metabolism	.42 (.07, .68)	.0211	.0403	.58 (.28, .77)	.0004	.0008
^d^ ^,^ ^e^KEGG butanoate metabolism	.40 (.05, .67)	.0270	.0497	.58 (.29, .77)	.0003	.0007
^c^KEGG cardiac muscle contraction	.38 (.02, .65)	.0378	.0654	.72 (.49, .85)	<.0001	.0003
^c^ ^,^ ^e^ ^,^ ^f^KEGG glycine serine and threonine metabolism	.53 (.21, .75)	.0023	.0066	.57 (.28, .77)	.0005	.001
^e^KEGG propanoate metabolism	.49 (.16, .72)	.005	.0124	.56 (.26, .76)	.0007	.0013
^d^KEGG pyrimidine metabolism	.39 (.03, .66)	.0323	.0574	.53 (.22, .74)	.0015	.0026
^c^KEGG renin angiotensin system	.59 (.30, .79)	.0004	.0014	.61 (.33, .79)	.0002	.0005
^c^KEGG steroid biosynthesis	.48 (.14, .71)	.0071	.0162	.72 (.50, .86)	<.0001	.0003
^c^REACTOME acyl chain remodelling of PG	.44 (.09, .69)	.0151	.0305	.45 (.13, .69)	.0083	.0118
^c^REACTOME ADP signalling through P2Y PURINOCEPTOR 1	.61 (.33, .80)	.0002	.0008	.53 (.23, .74)	.0013	.0023
^c^REACTOME ADP signalling through P2Y PURINOCEPTOR 12	.49 (.16, .72)	.0055	.0132	.66 (.40, .82)	<.0001	.0003
^c^REACTOME antigen processing cross presentation	.45 (.10, .70)	.0123	.0257	.72 (.50, .86)	<.0001	.0003
^c^REACTOME cellular response to chemical stress	.38 (.02, .65)	.0384	.0657	.71 (.48, .85)	<.0001	.0003
^d^REACTOME CHK1 CHK2 CDS1 mediated inactivation of cyclin B CDK1 complex	.52 (.20, .74)	.0028	.0077	.62 (.34, .79)	.0001	.0003
^c^ ^,^ ^d^REACTOME CILIUM assembly	.39 (.03, .65)	.0346	.0610	.37 (.02, .63)	.0391	.0471
^d^REACTOME cooperation of PDCL PHLP1 and TRIC CCT in G protein beta folding	.45 (.11, .70)	.0112	.0239	.64 (.37, .81)	<.0001	.0003
^d^REACTOME cooperation of prefoldin and TRIC CCT in ACTIN and tubulin folding	.47 (.14, .71)	.0074	.0167	.56 (.27, .76)	.0006	.0012
^c^REACTOME Creation of C4 and C2 activators	.61 (.32, .79)	.0002	.0008	.40 (.05, .65)	.0242	.0303
^d^REACTOME Cyclin D associated events in G1	.37 (.01, .64)	.0445	.0747	.67 (.43, .83)	<.0001	.0003
^c^REACTOME diseases of carbohydrate metabolism	.61 (.32, .79)	.0002	.0008	.64 (.37, .81)	<.0001	.0003
^d^REACTOME folding of actin by CCT TRIC	.43 (.08, .68)	.0174	.0342	.49 (.17, .71)	.0043	.0066
^c^REACTOME G beta gamma signalling through PI3KGAMMA	.61 (.32, .80)	.0002	.0008	.37 (.03, .64)	.0338	.0413
^d^REACTOME G protein activation	.58 (.27, .78)	.0006	.0020	.56 (.26, .76)	.0007	.0013
^d^REACTOME G protein beta gamma signalling	.54 (.23, .76)	.0016	.0047	.35 (.00, .62)	.0480	.0567
^d^REACTOME gap junction degradation	.49 (.15, .72)	.0058	.0136	.63 (.37, .81)	<.0001	.0003
^d^REACTOME glucagon type ligand receptors	.49 (.16, .72)	.0053	.0130	.59 (.30, .78)	.0003	.0007
^c^REACTOME gluconeogenesis	.51 (.18, .74)	.0035	.0090	.56 (.26, .76)	.0007	.0013
^d^REACTOME glutamate and glutamine metabolism	.55 (.23, .76)	.0014	.0043	.50 (.18, .72)	.0033	.0052
^c^REACTOME glutathione conjugation	.53 (.21, .75)	.0023	.0066	.63 (.36, .80)	<.0001	.0003
^d^REACTOME glycogen synthesis	.60 (.30, .79)	.0004	.0014	.74 (.53, .87)	<.0001	.0003
^c^REACTOME infection with *Mycobacterium tuberculosis*	.46 (.12, .71)	.0092	.0202	.44 (.10, .68)	.0120	.0163
^d^REACTOME LDL clearance	.45 (.11, .70)	.0121	.0255	.66 (.40, .82)	<.0001	.0003
^c^REACTOME metabolism of amino acids and derivatives	.40 (.05, .66)	.028	.0509	.68 (.43, .83)	<.0001	.0003
^f^REACTOME metabolism of folate and pterines	.60 (.30, .79)	.0004	.0014	.40 (.05, .65)	.0242	.0303
^f^REACTOME methylation	.52 (.20, .74)	.0026	.0072	.67 (.42, .83)	<.0001	.0003
^c^REACTOME NEF mediates down modulation of cell surface receptors by recruiting them to clathrin adapters	.37 (.02, .65)	.0410	.0693	.47 (.15, .70)	.0060	.0088
^d^REACTOME pentose phosphate pathway	.49 (.15, .72)	.0056	.0133	.53 (.23, .74)	.0013	.0023
^c^REACTOME protein folding	.57 (.26, .77)	.0008	.0026	.66 (.40, .82)	<.0001	.0003
^c^ ^,^ ^d^REACTOME signaling by robo receptors	.43 (.08, .68)	.0169	.0334	.60 (.32, .79)	.0002	.0005
^e^REACTOME synthesis of very long chain fatty acyl coas	.51 (.19, .74)	.0031	.0082	.79 (.61, .89)	<.0001	.0003
^c^REACTOME the role of NEF in HIV 1 replication and disease pathogenesis	.56 (.25, .77)	.0010	.0032	.47 (.14, .70)	.0065	.0094
^c^REACTOME Thromboxane signalling through TP receptor	.58 (.28, .78)	.0006	.0020	.65 (.39, .81)	<.0001	.0003
^d^ ^,^ ^e^REACTOME TP53 regulates metabolic genes	.42 (.07, .68)	.0209	.0403	.64 (.38, .81)	<.0001	.0003
^e^REACTOME translation	.38 (.03, .65)	.0352	.0613	.58 (.29, .77)	.0004	.0008
^c^REACTOME WNT5A dependent internalization of FZD2 FZD5 and ROR2	.55 (.24, .76)	.0013	.004	.57 (.28, .77)	.0005	.001
^d^WP amino acid metabolism	.53 (.20, .74)	.0024	.0068	.71 (.47, .85)	<.0001	.0003
^d^WP cholesterol metabolism with bloch and Kandutschrussell pathways	.63 (.35, .81)	.0001	.0005	.77 (.57, .88)	<.0001	.0003
^d^WP common pathways underlying drug addiction	.52 (.19, .74)	.003	.0081	.55 (.25, .75)	.0009	.0017
^e^WP metabolic reprogramming in colon cancer	.49 (.16, .72)	.0054	.0131	.58 (.29, .77)	.0003	.0007
^c^WP metapathway biotransformation phase I and II	.60 (.30, .79)	.0004	.0014	.73 (.51, .86)	<.0001	.0003
^d^WP Mitochondrial complex III assembly	.45 (.11, .70)	.0118	.025	.53 (.22, .74)	.0015	.0026
^d^WP Nanoparticle triggered regulated necrosis	.54 (.23, .76)	.0015	.0045	.42 (.08, .67)	.0170	.0219
^f^WP Onecarbon metabolism	.38 (.02, .65)	.0383	.0657	.44 (.11, .69)	.0105	.0145
^f^WP Onecarbon metabolism and related pathways	.53 (.22, .75)	.0019	.0056	.54 (.23, .75)	.0012	.0021
^c^ ^,^ ^d^WP purine metabolism and related disorders	.63 (.34, .80)	.0001	.0005	.60 (.31, .78)	.0002	.0005
^c^ ^,^ ^d^WP RALA downstream regulated genes	.60 (.31, .79)	.0003	.0012	.70 (.46, .84)	<.0001	.0003

^a^ For GSE73712 dataset, the five common lincRNAs-based risk score = (5-*LINC01089*) + (6-*LINC00482*) + (4-*LINC00893*) + (5-*LINC00649*) + (4-*LINC00265*) based on the identified five common lincRNAs, in Table 2 and their corresponding maximum values. For GSE174415 dataset, the five common lincRNAs-based risk score = (4-*LINC01089*) + (5-*LINC00482*) + (5-*LINC00893*) + (5-*LINC00649*) + (5-*LINC00265*) based on the identified five common lincRNAs, in Table 2 and their corresponding maximum values.

^b^ Cut-off values for multiple Pearson correlations were set as *p* < .05 and FDR<.10.

^c^ The category of immune, inflammation, and oxidative stress.

^d^ The category of cell cycle, apoptosis, autophagy, and cellular senescence.

^e^ The category of energy metabolism and protein synthesis.

^f^ The category of methylation.

## 4 Discussion

In the present study, we found that nine core molecular clock genes are dysregulated and are accompanied by the robust and significant decrease of five circadian lincRNAs in the placenta in sPTB vs term births across two independent datasets. We also found that these five circadian lincRNAs share more than 40% sequence identity, suggesting that they are very likely to share some functional similarities (Pearson, 2013). To our knowledge these findings for the first time suggest that both core molecular clock genes and lincRNAs in the placenta may be coordinately associated with sPTB. Some lncRNAs have been reported to be controlled by molecular clock genes (Qi et al., 2020) or regulate the transcriptional expression of molecular clock genes (Wang et al., 2020). However, the cause-effect relationship between the identified molecular clock genes and the five lincRNAs in the placenta in sPTB vs term is unknown. Both *LINC00893* and *LINC00265* have been linked to circadian rhythm pathways in cancers (Ge et al., 2019; Li et al., 2021). The five lincRNAs identified here have also been reported to be involved in proliferation, migration, invasion, apoptosis, inflammation, angiogenesis, and epithelial-mesenchymal transition in various cancer cells (Li et al., 2020; Zhang et al., 2020; Wang et al., 2021a; Wang et al., 2021b; Ou et al., 2021; Zhi et al., 2021). These cancer pathways partially overlap with our findings from the pathway analysis of these five circadian lincRNAs (see below for details). It must be mentioned that our PCA analysis reveals a substantial heterogeneity of the expression of these five circadian lincRNAs across individual samples in both groups in two datasets. Such heterogeneity might be related to the variations in time-of-day/time-of-year of the sample collection and/or placental locations of sampling as well as the heterogeneous sPTB subtypes.

The core molecular clock genes associated with the five circadian lincRNAs in our two datasets are not exactly the same (*NPAS2*, *NR1D1*, *NR1D2*, *PER3*, and *RORA* in GSE73712; *ARNTL*, *CRY1*, *NPAS3*, *PER2*, and *RORA* in GSE174415). This might be due to the following possible differences between datasets: 1) time of day for sample collection; 2) race/ethnicity (unknown in GSE73712 and African American in GSE174415); 3) definition of sPTB, 4) sampling regions of the placenta; and/or 5) inclusion/exclusion criteria of the participants. Interestingly, the *RORA* gene transcript was decreased in the sPTB group among both datasets. In mice, the *Rora* gene (a homolog of the human *RORA* gene) mediates the control of cell autonomous BMAL1 oscillations *via* a ROR/REV-ERB-response element (RORE)-dependent mechanism whereas the CLOCK:BMAL1 heterodimer drives rhythmic transcription of other core molecular clock genes *via* their binding to the E-box elements in the promoters of these target genes (Kondratov et al., 2003; Akashi and Takumi, 2005; Solt et al., 2011). Thus, the placental *RORA* gene might function as a gatekeeper to control the circadian rhythmic expressions of these sPTB-associated lincRNAs. Our results indicate that the *RORA* gene may be a robust target for investigating the circadian lincRNA-sPTB relationship and underlying mechanisms. Further, in our recent study (Zhou et al., 2022), we found that the placenta from women with preeclampsia presented with increased *CRY1* mRNA as well as reduced *NR1D2* and *PER3* mRNA. Therefore, there appears to be some degree of specificity in the change of placental molecular clock genes in different pregnancy complications.

Using the GSVA method, we observed that a total of 65 pathways are significantly enriched by the five circadian lincRNAs in the placenta and can be broadly grouped into immune/inflammation/oxidative stress, cell cycle/apoptosis/autophagy/cellular senescence as well as energy metabolism/protein synthesis- and/or methylation-related pathways. It is considered that among the major mechanisms underlying the etiology and/or pathogenesis of sPTB are immune responses and inflammation at the maternal-fetal interface (Romero et al., 2014; Brien et al., 2019; Goldstein et al., 2020; Green and Arck, 2020; Humberg et al., 2020; Parris et al., 2021). sPTB has also been linked to placental oxidative stress (Chang et al., 2013; Menon, 2014; Polettini et al., 2014; Perrone et al., 2016; Saroyo et al., 2021), cellular senescence (Cha and Aronoff, 2017; Menon et al., 2017; Saroyo et al., 2021), apoptosis (Tarquini et al., 2018; Kovács et al., 2020), autophagy (Agrawal et al., 2015; Cao et al., 2016), methylation (Tilley et al., 2018; Mani et al., 2019; Schuster et al., 2019; Li et al., 2020; Wang et al., 2022), and energy metabolism (Elshenawy et al., 2020; Lien et al., 2020; Lien et al., 2021). In addition, there is evidence that protein synthesis is inversely related to birth weight and gestational age (Johnson and Metcoff, 1986). Taken together, our results suggest that the identified five circadian lincRNAs might be involved in placental intertwined pathophysiological processes leading to sPTB. The 65 identified pathways warrant further investigation to elucidate the functions of the five placental circadian lincRNAs in relation to sPTB and possible therapeutic targets for prevention of sPTB. Larger clinical and experimental studies are needed to further clarify the relationships among molecular clock genes, lincRNAs, and sPTB as well as the underlying mechanisms.

Major strengths of our study are the robust bioinformatics approach and the use of two independent datasets to improve the generalizability of the results. Among the limitations are a lack of information about the time-of-day/time-of-year of the sample collection, incomplete list of the lncRNAs in both datasets, relatively small sample size (20 sPTBs in GSE73712, 16 sPTBs in GSE174415), no detailed pathological information about the placental samples, non-available maternal demographics (race, age, and parity) in one or two datasets, different sPTB definitions, different placental locations of sampling in the two datasets, bulk RNAseq data only (not single-cell RNAseq data), possible bias introduced by no labor in term in the dataset GSE73712, and a lack of experimental validation of the identified circadian lincRNAs associated with sPTB and their enriched biological pathways.

In conclusion, the decrease in levels of five circadian lincRNA transcripts in sPTB placentas is associated with the altered pathways involving cellular senescence, apoptosis, autophagy, immune response, inflammation, oxidative stress, energy metabolism, protein synthesis, and methylation. Further exploration of these five circadian genes-correlated lincRNAs and their associated pathways may improve our understanding of the pathogenesis of sPTB and provide novel insights into the development of potentially more effective and specific therapeutic targets to prevent sPTB.

## Data Availability

Publicly available datasets were analyzed in this study. This data can be found here: https://www.ncbi.nlm.nih.gov/geo/query/acc.cgi?acc=GSE73712 NCBI GEO GSE174415.
