# Supplementary material for: Placental circadian lincRNAs and spontaneous preterm birth
Source: Front Genet. 2023 Jan 11;13:1051396. doi: 10.3389/fgene.2022.1051396 (PMC9874002; doi:10.3389/fgene.2022.1051396)
Supplement: Supplementary file 3 [file DataSheet1.pdf]

A

B

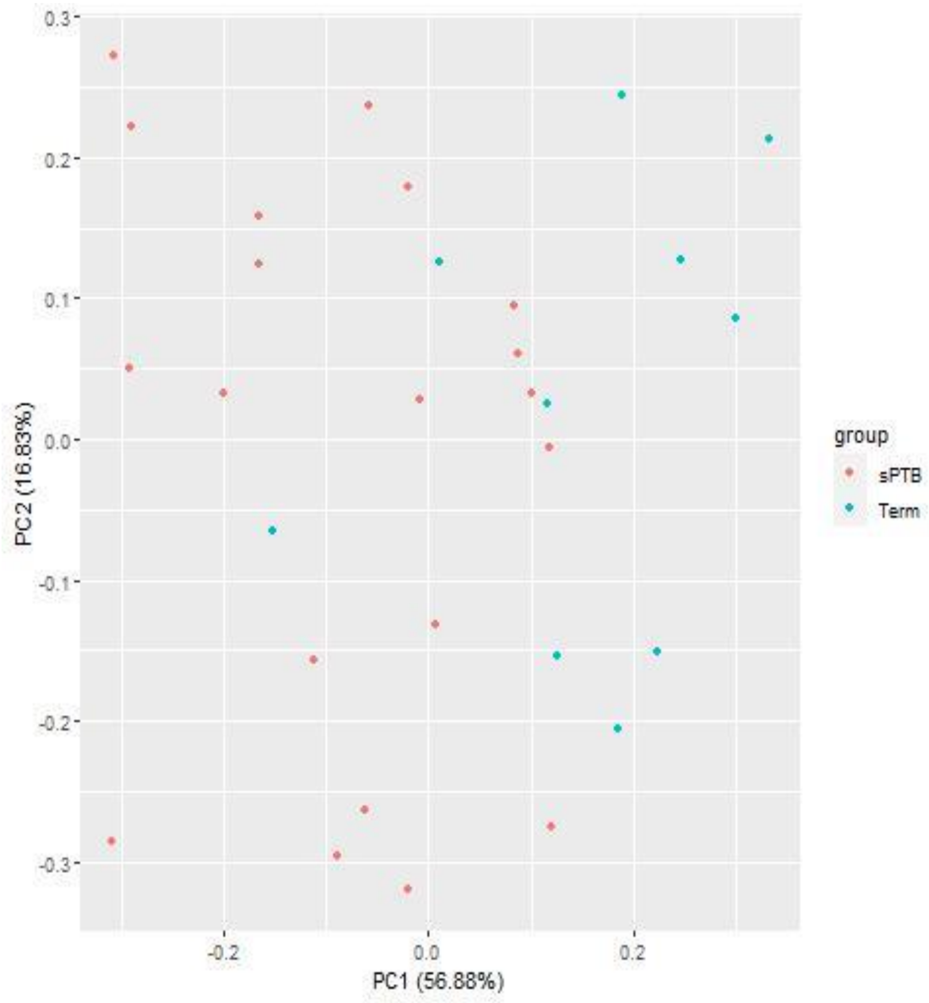

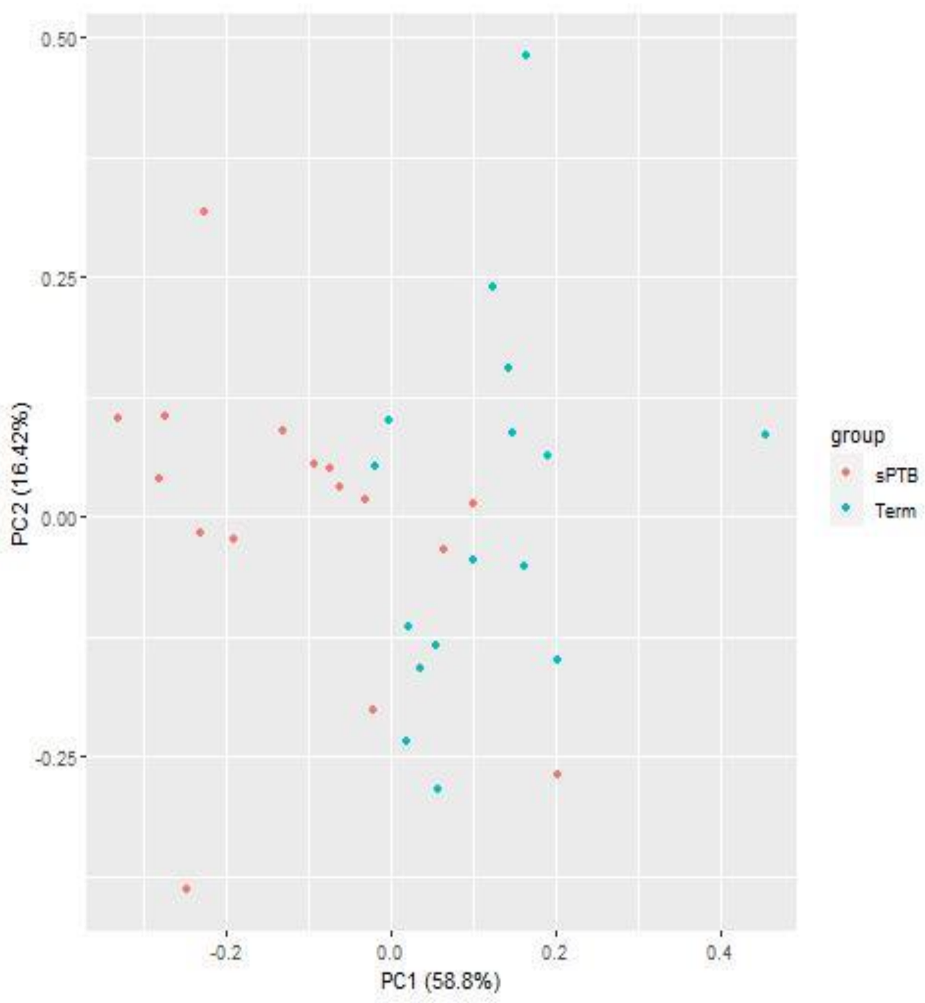

Figure S1. PCA analysis of 5 circadian lincRNAs to visualize the clusters due to the differences in 5 circadian lincRNAs between sPTB and term births and the corresponding variability. A is for GSE73712 and B for GSE174415.
